# Lemmel Syndrome as a Rare Cause of Prolonged Right Hypochondrial Pain: A Case Report

**DOI:** 10.7759/cureus.20093

**Published:** 2021-12-01

**Authors:** Ayman Z Azzam, Tuqa A Alsinan, Ghader A Alrebeh, Tahirah Alhaider, Lara J Alnaqaeb, Tarek M Amin

**Affiliations:** 1 Department of General Surgery, Faculty of Medicine, Alexandria University, Alexandria, EGY; 2 Department of Surgical Oncology, Oncology Centre, King Faisal Specialist Hospital and Research Centre, Riyadh, SAU; 3 Department of Surgery, King Saud Medical City, Riyadh, SAU; 4 College of Medicine, Alfaisal University, Riyadh, SAU

**Keywords:** magnetic resonance cholangiopancreatography (mrcp), endoscopic retrograde cholangiopancreatography (ercp), periampullary dilatation, duodenal diverticulum, hypochondrial pain, lemmel syndrome

## Abstract

Lemmel syndrome is a rare cholestatic disease caused by a periampullary duodenal diverticulum (PAD) compressing the common bile duct (CBD) or pancreatic duct, which results in acute abdominal pain and/or obstructive jaundice in the absence of other pathology explaining the symptoms. It can be easily misdiagnosed unless carefully detected by abdominal ultrasound (US), barium studies, computed tomography (CT) scan, esophagogastroduodenoscopy (EGD), magnetic resonance cholangiopancreatography (MRCP), and endoscopic retrograde cholangiography (ERCP), which is also the treatment modality of choice. We herein report a case of a 62-year-old male presenting with prolonged hypochondrial pain. He was diagnosed with Lemmel syndrome after performing US, barium meal, CT scan, EGD, and MRCP that was managed successfully by ERCP with sphincterotomy and stent placement.

## Introduction

Lemmel syndrome was first defined by Lemmel in 1934 as a diverticulum of the periampullary duodenum causing obstructive jaundice in the absence of choledocholithiasis or neoplasm [1]. The duodenal diverticulum is most commonly a pseudo-diverticulum that is made of a sac-like outpouching of the mucosa and submucosa lacking a complete muscularis layer [2-4]. It is mostly found in the second part of the duodenum, 2-3 cm near the ampulla of Vater. With advancing age, the incidence of the periampullary duodenal diverticulum (PAD) increases [3-4]. Up to 22% of cases are incidentally found as they are usually asymptomatic, while less than 10% are complicated with inflammation presenting with obstructive symptoms including abdominal pain and jaundice [2-4]. This presentation makes Lemmel syndrome easily misdiagnosed with other benign or malignant abnormalities in the periampullary region [5]. Around 1-5% of cases only present with diverticular complications such as obstruction, diverticulitis, hemorrhage, and perforation [3]. The diagnosis is reached by imaging modalities such as abdominal ultrasound (US), barium studies, CT scan, esophagogastroduodenoscopy (EGD), and magnetic resonance cholangiopancreatography (MRCP) [2-6]. Conservative management is the mainstay of care in these patients by endoscopic retrograde cholangiography (ERCP) with sphincterotomy and stent placement [4,5,7-10]. Surgical intervention may be warranted in cases of complications, recurrence, or conservative measures failure [2].

## Case presentation

**Figure 1:**
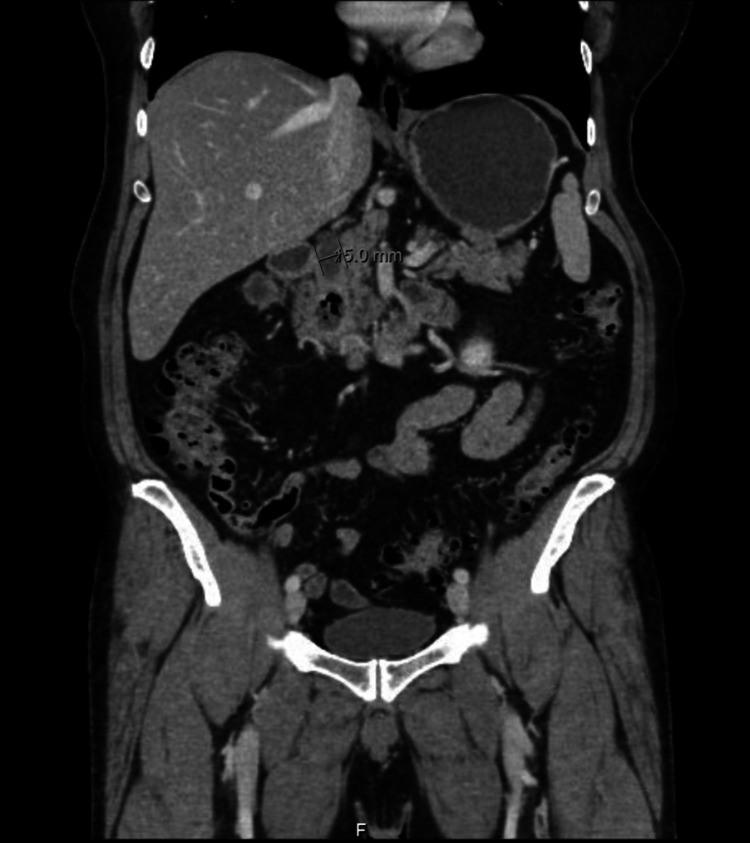
CT abdomen and pelvis (coronal view) showing a duodenal diverticulum measuring 5 mm and arising from the second part of the duodenum.

A 62-year-old previously healthy male presented to the surgical oncology clinic with on and off right hypochondrial pain for two years with no other symptoms. The rest of his history was unremarkable. Physical examinations were all within the normal limits including negative Murphy's sign. All his labs were in the normal range including complete blood count, hepatic profile, renal profile, and serum electrolytes. He was referred from his local hospital after being diagnosed with a duodenal diverticulum measuring 5 mm in diameter with common bile duct (CBD) dilatation associated with gallstones in the absence of cholecystitis as shown on CT scan of the abdomen and pelvis (Figure 1), MRI, and EGD.

**Figure 2:**
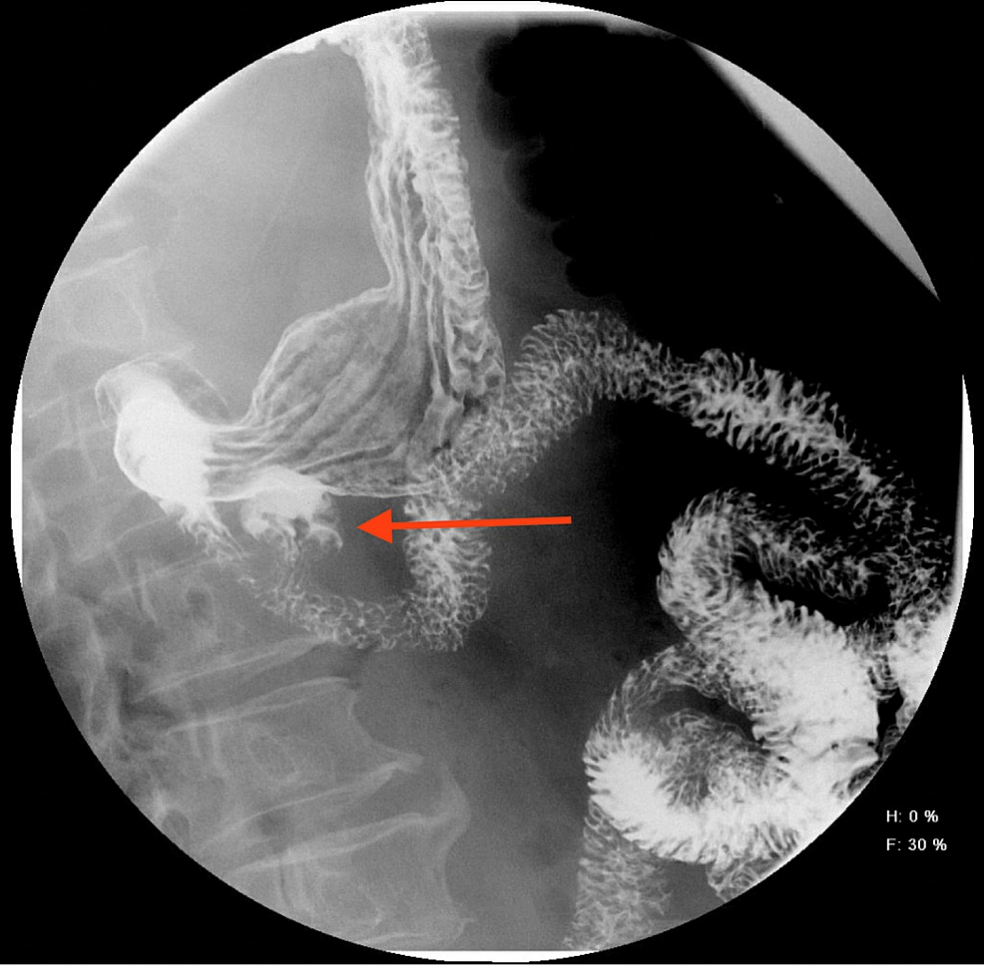
Barium meal showing PAD (arrow) in the second part of the duodenum. PAD, periampullary duodenal diverticulum.

Furthermore, a contrast barium meal was ordered, which showed a duodenal diverticulum in the second part (Figure 2).

**Figure 3:**
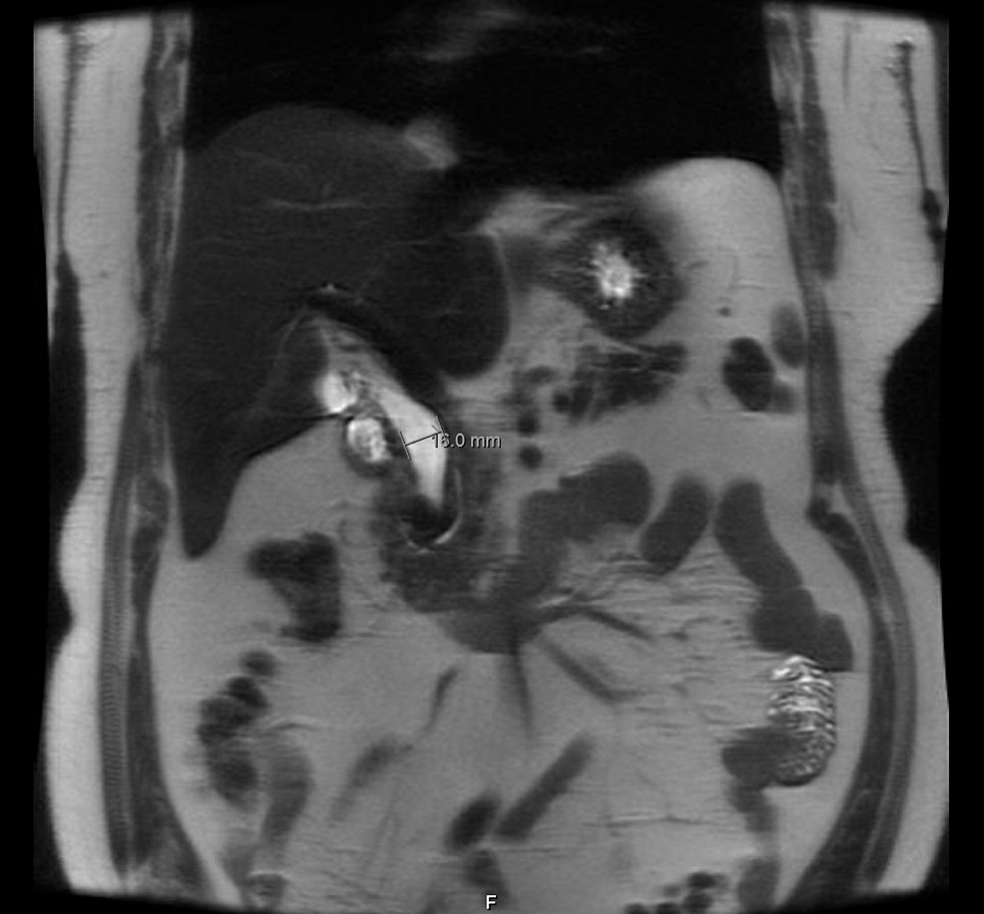
MRCP showing dilatation of CBD measuring 16 mm in diameter. MRCP, magnetic resonance cholangiopancreatography; CBD, common bile duct.

MRCP was done, which showed a dilated CBD measuring up to 16 mm due to the effect of PAD strictly adjacent to its distal portion that is associated with moderate hepatic steatosis and presence of gallstones without evidence of cholecystitis (Figure 3).

Based on the clinical presentation and imaging findings, the diagnosis of Lemmel syndrome was reached. The patient was referred to the gastroenterology team for ERCP, which showed a dilated CBD. Management with sphincterotomy and stent placement was done, which relieved the patient’s pain completely leading to his discharge on regular follow-up with MRCP and labs every six months.

## Discussion

Lemmel syndrome was first defined as a PAD causing obstructive jaundice in the absence of choledocholithiasis or neoplasm [1]. The duodenal diverticulum is an incidental finding in most cases as a pseudodiverticulum in the second part occasionally in the elderly [2-4]. In rare events, complications develop leading to abdominal pain and/or obstructive symptoms [3]. For that, a number of cases were reported in the literature highlighting the importance of considering such a disease to overcome misdiagnosis.

From 2005 to 2017, several cases of Lemmel syndrome have been reported. In these patients who presented to the emergency department, signs and symptoms of biliary obstruction such as acute abdominal pain, unintentional weight loss, generalized fatigue, and active infection were encountered. Most of them were diagnosed based on imaging findings of MRCP and ERCP with contrast. They were managed conservatively by antibiotics and nasogastric tube decompression [4,5].

Meanwhile, around four cases were reported in Europe recently between 2019 and 2020. In these cases, the presentation was mainly acute recurrent abdominal pain, which required a visit to emergency services. Diagnosis of Lemmel syndrome was made based on CT findings, which showed a typical PAD with associated infection. Patients improved with administration of antibiotics, placement of plastic biliary stent by ERCP, and drainage of the obstruction, respectively [3,4,6].

In addition, a retrospective and two case reports were conducted in Europe and the United States between 2006 and 2019. They studied the effectiveness of interventional management in patients diagnosed with Lemmel syndrome. It was shown that endoscopic sphincterotomy with multiple balloon sweeps results in a better outcome and fewer complications [7,8,10]. Moreover in 2017, a rare case of complicated Lemmel syndrome presented with signs of jaundice and pancreatitis that underwent conservative management with nil per os (NPO), antibiotics, intravenous fluid replacement, strict observation, and discharged on follow up. This patient had a recurrence after two months requiring total resection of the duodenal diverticulum [2].

## Conclusions

Lemmel syndrome should be considered as a differential diagnosis in patients presenting with acute abdominal pain and/or obstructive jaundice, even though it is a rare cause. Confirming the diagnosis mandates imaging modalities such as CT scan being a non-invasive, rapid, and specific choice. Barium contrast and MRCP may be needed to confirm the diagnosis or exclude other causes of acute abdominal pain and/or obstructive jaundice. Conservative management by ERCP with sphincterotomy and stent placement is the mainstay treatment in Lemmel syndrome. However, close follow-up and surgical intervention may be considered in severe cases.
